# Phenotypic discordance in rifampicin resistance detection among *Mycobacterium tuberculosis* isolates from China: insights from whole-genome sequencing and a structured literature review

**DOI:** 10.1128/spectrum.03261-25

**Published:** 2026-07-06

**Authors:** Ya-Li Chen, Cui-Ping Guan, Yun-Feng Deng, Mao-Shui Wang, Zhong-Fa Zhang

**Affiliations:** 1 Department of Lab Medicine, Public Health Clinical Center Affiliated to Shandong University 442536, Jinan, China; 2 Katharine Hsu International Research Center of Human Infectious Diseases, Public Health Clinical Center Affiliated to Shandong University 442536, Jinan, China; 3 Department of Respiratory Medicine, Public Health Clinical Center Affiliated to Shandong University 442536, Jinan, China; ICON plc, London, United Kingdom

**Keywords:** *Mycobacterium tuberculosis*, rifampicin, drug susceptibility testing, whole-genome sequencing, *rpoB*

## Abstract

Phenotypic drug susceptibility testing (pDST) for rifampicin (RIF) resistance in *Mycobacterium tuberculosis* (MTB) can yield discordant results between solid and liquid culture methods, complicating accurate detection of drug-resistant tuberculosis (DR-TB). We evaluated RIF pDST concordance in 265 clinical isolates (July 2016 through June 2023) tested on Lowenstein-Jensen (L-J) medium and the Mycobacteria Growth Indicator Tube (MGIT) 960 system. Discordant isolates underwent repeat testing and whole-genome sequencing (WGS). Genotypic data were interpreted with validated pipelines. A structured literature review synthesized published data on discordant RIF phenotypes and associated *rpoB* variants. Overall, 11.7% (31/265) of the isolates were discordant, with 17 L-J resistant/MGIT-susceptible, and 14 the reverse. Discrepancies persisted on retesting in 59% (16/27). WGS of 10 sequenced discordant isolates showed that all harbored *rpoB* mutations associated with rifampicin resistance, largely within the rifampicin resistance-determining region (RRDR), including Leu452Pro (5/10) and Asp435Tyr (1/10), plus compound patterns (Asp435Tyr + Asn437 Asp; Leu430Pro + Asn437 Asp; Met434Val + Asp435 Gly). All isolates belonged to the East Asian/Beijing lineage and shared a synonymous *rpoB* Ala1075Ala; one had a synonymous *rpoA* Tyr341Tyr. Putative compensatory *rpoC* mutations (Lys71Asn, Glu49Gly) were detected. The literature review corroborated frequent association of Leu452Pro, Asp435Tyr, Leu430Pro, and His445 variants with inter-method discordance and previously reported low-level RIF resistance near critical concentrations. RIF pDST discordance exceeding 10% reflects inherent methodological limits and the variable phenotypic expression of specific *rpoB* mutations, notably Leu452Pro, which may fall below MGIT detection thresholds. In high-suspicion or phenotypically discordant cases, WGS may improve resistance classification and help inform treatment decisions and transmission control.

**IMPORTANCE:**

Accurate detection of RIF resistance underpins rapid initiation of effective TB therapy and containment of transmission. However, more than 1/10 MTB isolates in our cohort produced reproducible, method-dependent discordance between solid (L-J) and liquid (MGIT) pDST tests. Such discordance risks misclassifying genuinely resistant strains as susceptible, especially when low-level resistance mutations (notably *rpoB* Leu452Pro and certain Asp435 and Leu430 variants) sit near critical concentration thresholds used in automated liquid systems. Reliance on a single phenotypic platform may therefore delay appropriate multidrug-resistant TB regimens and foster ongoing spread. Among the sequenced discordant isolates, WGS identified resistance-associated *rpoB* mutations in all cases, with putative compensatory *rpoC* mutations detected in some isolates, supporting its potential value as an adjudication tool. Integrating rapid genotypic methods when phenotypic results conflict, or when clinical suspicion remains high, may improve regimen selection and laboratory quality assurance.

## INTRODUCTION

According to the Global Tuberculosis Report 2025 (1), an estimated 390,000 individuals (95% uncertainty interval [UI]: 360,000–430,000) developed multidrug-resistant or rifampicin-resistant tuberculosis (MDR/RR-TB) in 2024. However, only 42% of these cases were diagnosed and commenced on treatment, with a treatment success rate of just 71%. Rifampicin (RIF), a cornerstone of first-line tuberculosis (TB) therapy, serves as a critical marker for MDR/RR-TB. Therefore, the rapid and reliable detection of RIF resistance is essential for effective diagnosis, treatment guidance, and containment of drug-resistant TB (DR-TB). Mutations in the *rpoB* gene are the principal genetic determinants of RIF resistance, accounting for over 95% of documented cases.

RIF-susceptibility testing is typically conducted using either phenotypic drug susceptibility testing (pDST) or genotypic drug susceptibility testing (gDST). Traditionally, pDST, which is based on the growth of *Mycobacterium tuberculosis* (MTB) in media containing a critical concentration (CC) of RIF, has been considered the gold standard for resistance detection (2). However, recent evidence underscores notable limitations of pDST, especially in identifying borderline *rpoB* mutations. These mutations result in modest reductions in bacterial fitness (2, 3), often producing resistance levels that fall between full susceptibility and high-level resistance, making them difficult to detect accurately by pDST (4–6). The Mycobacteria Growth Indicator Tube (MGIT) system, in particular, frequently fails to detect borderline mutations, often producing false-susceptible results (7–9). Similarly, the Lowenstein-Jensen (L-J) culture method also exhibits limited sensitivity for detecting borderline-resistant strains, although its sensitivity may be enhanced by prolonged incubation (10). While the overall agreement between MGIT and L-J is relatively high (kappa = 0.79), persistent inconsistencies remain (11). These discrepancies are largely attributed to differences in each method’s ability to detect low-level resistance and the overlap in minimum inhibitory concentrations (MICs) between susceptible and borderline-resistant isolates (12–15). These limitations highlight the urgent need for complementary molecular diagnostics and careful interpretation of pDST results, particularly in cases where borderline *rpoB* mutations are suspected, to enhance the accuracy of RIF resistance detection.

Given these challenges, this study aimed to systematically evaluate RIF susceptibility results obtained using solid (L-J) and liquid (MGIT) culture methods in MTB isolates from China. Isolates exhibiting discordant pDST results were subjected to whole-genome sequencing (WGS) to elucidate the underlying mechanisms of resistance. Furthermore, a structured literature review was conducted to synthesize fragmented evidence on mutations associated with such discordant RIF resistance profiles, thereby enhancing the clinical relevance and applicability of the study findings. Through this integrated approach, the study aimed to evaluate the potential utility of WGS for clarifying discordant RIF susceptibility results and to summarize published evidence on mutations associated with such discrepancies. Ultimately, this work seeks to contribute to improved interpretation of discordant RIF resistance and support more accurate diagnosis and management of RR-TB.

## MATERIALS AND METHODS

### Study design

Between July 2016 and June 2023, a total of 265 MTB isolates from individual patients were concurrently evaluated for RIF resistance using L-J solid medium (Zhuhai Beisuo Co., China) and the BACTEC MGIT 960 liquid culture system (Becton Dickinson, USA) at the Public Health Clinical Center Affiliated to Shandong University. To minimize variability in pDST and reduce the likelihood of misclassification due to unstable phenotypic results, all isolates showing discordant RIF resistance results between the two methods underwent repeat testing. Isolates selected for WGS were those whose repeat pDST results were concordant with their respective initial results, and in practice, these isolates remained discordant between the two phenotypic methods. Additionally, a structured literature review was conducted to summarize *rpoB* gene mutations in isolates with discrepant RIF susceptibility between solid and liquid culture methods.

### RIF-susceptibility testing

Stored MTB isolates were subcultured on L-J medium and harvested after 2–3 weeks for susceptibility testing. For the solid culture method, L-J medium supplemented with RIF at a critical concentration of 40 µg/mL was used, with drug-free L-J medium serving as the control. After 4 weeks of incubation, RIF susceptibility was determined based on visible bacterial growth. For the liquid culture method, the BACTEC MGIT 960 system was employed according to the manufacturer’s instructions, using a critical RIF concentration of 1 µg/mL. MGIT tubes were incubated at 37°C, and bacterial growth was automatically monitored via oxygen consumption.

Isolates showing discordant results between the two methods underwent repeat testing. Isolates with stable repeat pDST results were selected for WGS analysis.

### Sequencing analysis

Genomic DNA was extracted from heat-inactivated MTB cultures using a commercial DNA extraction and purification kit (D4015-02, Omega Inc., USA), following the manufacturer’s instructions. DNA quality was assessed by Qubit fluorometric quantification (Thermo Fisher Scientific, USA) and agarose gel electrophoresis, and samples were stored at −80°C until use.

DNA libraries were prepared using the TruSeq DNA Sample Prep Kit (FC-121-4001, Illumina, San Diego, CA). DNA fragmentation was performed using dsDNA Fragmentase (NEB, M0348S) at 37°C for 30 min. Paired-end sequencing was carried out on the Illumina NovaSeq 6000 platform (Illumina, USA) using standard protocols. Sequencing was conducted by Jinan Jinyu Medical Laboratory Co., Ltd.

Raw sequencing reads were processed to remove adapters using Cutadapt (v1.9) and trimmed for quality using Fqtrim (v0.9.4) with a sliding-window algorithm. Read quality was assessed using FastQC (v0.10.1). Cleaned reads were aligned to the MTB H37Rv reference genome (GenBank accession no. NC_000962.3) via the Galaxy platform (usegalaxy.eu) (16). Variant calling was performed using Snippy (v4.6.0), which incorporates BWA for alignment and FreeBayes for the detection of single-nucleotide polymorphisms (SNPs) and insertions/deletions (Indels). Variants were filtered with TB Variant Filter (v0.4.0) to enhance specificity. MTB lineages, sub-lineages, and resistance mutations, including SNPs, indels, and frameshifts, were identified using *TBProfiler* (v6.2.1, TBDB database v82777ea) (17), and further validated with *Mykrobe* (v0.10.0) using the NC_000962.3 reference genome (18). All sequencing data have been deposited in the NCBI Sequence Read Archive under accession number PRJNA1108515.

### Structured literature review

A comprehensive literature search was conducted using PubMed, Embase, Web of Science, and Scopus to identify studies published before July 1, 2025, without language or publication-type restrictions. Studies were eligible if they reported discordant RIF resistance results between solid and liquid culture methods and documented the associated *rpoB* gene mutations. The detailed search strategy is provided in Supplemental material.

## RESULTS

### pDST results

**Fig 1:**
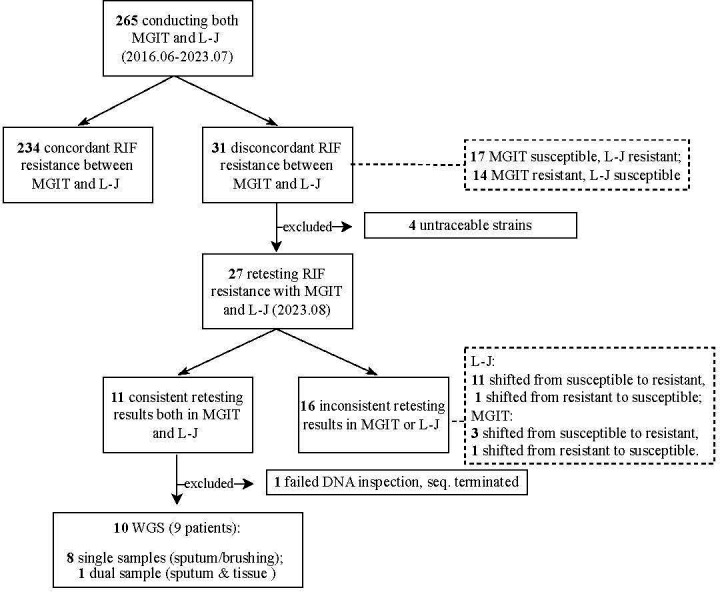
Flowchart illustrating the sample selection workflow Abbreviations: L-J, Lowenstein-Jensen; MGIT, Mycobacteria Growth Indicator Tube; WGS, whole-genome sequencing; RIF, rifampicin.

Figure 1 outlines the workflow for sample selection. Between July 2016 and June 2023, 265 MTB isolates were tested in parallel for RIF resistance using both L-J solid medium and the MGIT liquid culture systems. Among these, 31 isolates exhibited discordant results: 17 were RIF-susceptible by MGIT but resistant by L-J, while 14 were susceptible by L-J but resistant by MGIT. Four isolates were untraceable, and the remaining 27 underwent repeat DST using both methods. The detailed results of initial and repeat testing, including the direction of phenotype changes and isolates with unchanged results, are summarized in Table S1.

Upon retesting, 16 isolates exhibited results that differed from their initial classification: 11 isolates converted from susceptible to resistant, and 1 from resistant to susceptible (L-J method); 3 isolates shifted from susceptible to resistant, and 1 from resistant to susceptible (MGIT method). Eleven strains whose repeat pDST results were concordant with their respective initial results were selected for WGS, of which one failed quality control. Thus, 10 isolates were successfully sequenced. WGS showed that all sequenced discordant isolates harbored *rpoB* mutations associated with RIF resistance. Among the nine patients from whom these isolates were obtained, eight were diagnosed with pulmonary tuberculosis, and five had a history of previous TB treatment. No cases of treatment failure were observed.

### Genetic findings

All patients from whom the sequenced discordant isolates were obtained were diagnosed with MDR/XDR-TB. Genomic analysis using the *TBProfiler* pipeline identified all isolates as belonging to the East Asian/Beijing lineage. Of these, 80% (*n* = 8) were classified as sublineage L2.2.1, and 20% (*n* = 2) as L2.2.2. Spoligotyping confirmed that nine isolates (90%) belonged to the Beijing family ST1, while one (10%) isolate was identified as ST190 (Table S2). In addition, no evidence of mixed infection was detected. Additional genomic analysis was performed using the *Mykrobe* pipeline, and the comparative results from *TBProfiler* and *Mykrobe* are presented in Table S2.

All 10 sequenced isolates exhibited phenotypic discordance in RIF susceptibility, characterized by susceptibility on MGIT and resistance on L-J medium. This discrepancy was consistently associated with the presence of mutations in the *rpoB* gene, frequently accompanied by additional mutations in *rpoC* and *rpoA*. Genotypic analysis revealed both single and complex missense mutations within the 81 bp rifampicin resistance-determining region (RRDR) of *rpoB*. The most common single *rpoB* mutation was Leu452Pro (CTG→CCG), present in five isolates (50%). One isolate (10%) carried the Asp435Tyr (GAC→TAC) mutation.

**Fig 2:**
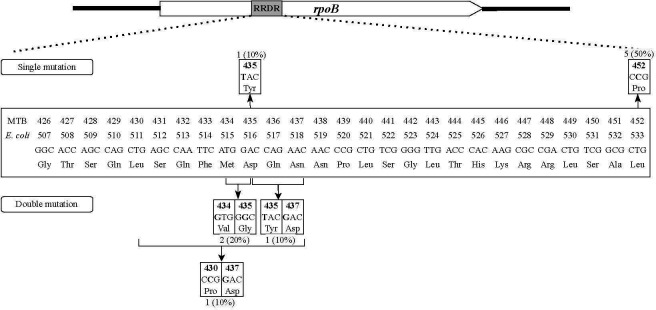
Distribution of mutations in the RRDR of the *rpoB* gene The original sequence of the *rpoB* gene is shown in the central box. An arrow highlights a specific region containing the mutated codons and their corresponding amino acid substitutions. For each codon, the frequency and percentage of isolates exhibiting the mutation are indicated next to the respective amino acid. Abbreviations: RRDR, rifampicin resistance-determining region.

Several isolates harbored complex mutation profiles. Isolate A5 harbored double missense mutations in *rpoB* (Asp435Tyr and Asn437Asp) and *rpoC* (Lys71Asn). Isolate A3 had combined *rpoB* mutations Leu430Pro (CTG→CCG) and Asn437Asp (AAC→GAC). Isolates A8 and A9 shared a dual mutation profile: Met434Val (ATG→GTG) and Asp435Gly (GAC→GGC). In isolate A1, Leu452Pro in *rpoB* was accompanied by Glu49Gly in *rpoC*. All isolates carried a synonymous mutation in *rpoB* (Ala1075Ala), and one isolate (A6) additionally carried a synonymous *rpoA* mutation (Tyr341Tyr; Table 1). The distribution of RRDR mutations is illustrated in Fig. 2.

**TABLE 1:** Summary of rifampicin discordance in drug susceptibility testing^*c*^

Clinical characteristics									Resistance to rifampicin			Variant characteristics			
Patient ID	Gender	Age (Years)	HIV status	Disease	Category	Outcomes	Specimen	SRA accession	L-J	MGIT	WGS	Gene	Type	Change	Confidence
A1	Male	51	Neg	PTB	Relapse	Completed	Sputum	SRR28923841	R	S	R	*rpoB*	missense variant	Leu452Pro^*a*^	Assoc w R
												*rpoC*	missense variant	Glu49Gly	Uncert. Sig.
												*rpoB*	synonymous variant	Ala1075Ala	Not assoc w R
A2	Male	20	Neg	PTB	Relapse	Completed	Sputum	SRR28923840	R	S	R	*rpoB*	missense variant	Leu452Pro^*a*^	Assoc w R
												*rpoB*	synonymous variant	Ala1075Ala	Not assoc w R
A3	Female	48	Neg	PTB	Relapse	Completed	Sputum	SRR28923839	R	S	R	*rpoB*	missense variant	Leu430Pro^*a*^	Assoc w R
												*rpoB*	missense variant	Asn437Asp^*b*^	Assoc w RI
												*rpoB*	synonymous variant	Ala1075Ala	Not assoc w R
A4	Male	37	Neg	PTB	Relapse	Completed	Sputum	SRR28923838	R	S	R	*rpoB*	missense variant	Leu452Pro^*a*^	Assoc w R
												*rpoB*	synonymous variant	Ala1075Ala	Not assoc w R
A5	Male	48	Neg	PTB	New case	Completed	Sputum	SRR28923837	R	S	R	*rpoB*	missense variant	Asp435Tyr^*a*^	Assoc w R
												*rpoB*	missense variant	Asn437Asp^*b*^	Assoc w RI
												*rpoC*	missense variant	Lys71Asn	Uncert. Sig.
												*rpoB*	synonymous variant	Ala1075Ala	Not assoc w R
A6	Male	33	Neg	PTB	New case	Cured	Sputum	SRR28923836	R	S	R	*rpoB*	missense variant	Asp435Tyr^*a*^	Assoc w R
												*rpoB*	synonymous variant	Ala1075Ala	Not assoc w R
												*rpoA*	synonymous variant	Tyr341Tyr	Not assoc w RI
A7	Male	25	Neg	PTB	Relapse	Cured	Brushing	SRR28923835	R	S	R	*rpoB*	missense variant	Leu452Pro^*a*^	Assoc w R
												*rpoB*	synonymous variant	Ala1075Ala	Not assoc w R
A8	Female	54	Neg	EPTB	New case	Completed	Tissue	SRR28923834	R	S	R	*rpoB*	missense variant	Met434Val^*b*^	Assoc w RI
												*rpoB*	missense variant	Asp435Gly	Assoc w RI
												*rpoB*	synonymous variant	Ala1075Ala	Not assoc w R
A9							Sputum	SRR28923833	R	S	R	*rpoB*	missense variant	Met434Val^*b*^	Assoc w RI
												*rpoB*	missense variant	Asp435Gly^*b*^	Assoc w RI
												*rpoB*	synonymous variant	Ala1075Ala	Not assoc w R
A10	Male	53	Neg	PTB	New case	Completed	Sputum	SRR28923832	R	S	R	*rpoB*	missense variant	Leu452Pro^*a*^	Assoc w R
												*rpoB*	synonymous variant	Ala1075Ala	Not assoc w R

^*a*^ Borderline rifampicin-resistance mutations.

^*b*^ Expert rule: Non-silent variants in RRDR of *rpoB*.

^*c*^ Neg, negative; ND, not detected; PTB, pulmonary tuberculosis; EPTB, extrapulmonary tuberculosis; L-J, lowenstein-Jensen; MGIT, Mycobacteria Growth Indicator Tube; Xpert, Xpert MTB/RIF; LPA, line probe assay; WGS, whole genome sequencing; R, resistant; S, susceptible; I, indeterminate; Assoc w R, associated with resistance; Assoc w RI, associated with resistance-interim; Not Assoc w R, not associated with resistance; Not Assoc w RI, not associated with resistance-interim; Uncert. Sig., uncertain significance.

### Findings from structured literature review

Our structured literature review examined phenotypic RIF susceptibility discrepancies in MTB isolates with *rpoB* mutations. Considerable variability was observed across different culture systems, including L-J, Middlebrook 7H10, and MGIT. Across both solid and liquid media, the most frequently implicated *rpoB* mutations were Asp435Tyr (33.73%), Leu452Pro (20.48%), Leu430Pro (11.65%), and His445 cluster mutations (21.29%).

When comparing L-J and MGIT, discordant results were frequently associated with Asp435Tyr (35.87%), Leu452Pro (21.52%), Leu430Pro (10.76%), and His445 (20.18%). For Middlebrook 7H10 versus MGIT, discrepancies primarily involved Leu430Pro (19.23%), Asp435Tyr (15.38%), and His445 (30.77%). Among the limited number of studies reporting data at both MGIT critical concentrations, lowering the RIF critical concentration from 1.0 µg/mL to 0.5 µg/mL did not appear to fully eliminate discordance for several commonly implicated *rpoB* mutations, including Asp435Tyr, Leu452Pro, Leu430Pro, and His445 mutations. However, this observation should be interpreted cautiously because the number of directly comparable studies was small.

The recurrent identification of these key *rpoB* mutations across diverse geographic regions and time periods supports their importance in interpreting discordant RIF susceptibility. Notably, these mutations have been identified in studies from Belgium (2009, 2013, and 2019), the United States (2014 and 2021), and Pakistan (2024 and 2025). Moreover, rare mutations such as Ser428Ile and Ile59Thr, along with complex compound mutations (e.g., Leu430Pro + Met434 Ile, Gln436His + Asn437 deletion), further highlight the intricate genetic basis of phenotypic variability in MTB drug resistance (Table S3).

Although lineage information was available for only a minority of the included studies, the reported discordant isolates were distributed across multiple genetic backgrounds, including Beijing (Lineage 2), Central Asian (Lineage 3), and Euro-American (Lineage 4) strains, suggesting that such discordance is not restricted to a single lineage background, although the limited availability of lineage-level data precludes firm conclusions regarding lineage effects.

## DISCUSSION

RIF is a cornerstone drug in TB treatment, and resistance to RIF serves as a critical marker for MDR-TB; however, reliable detection of RIF resistance remains a major diagnostic challenge. In this study, 265 MTB isolates which were tested for RIF susceptibility using solid (L-J) and liquid (MGIT 960) culture methods were re-evaluated. Discordant results occurred in 11.7% (31/265) of cases, with 17 isolates susceptible by MGIT but resistant by L-J, and 14 showing the reverse. WGS of 10 sequenced discordant isolates identified *rpoB* mutations previously associated with low-level rifampicin resistance, notably Leu452Pro (50%) and Asp435Tyr (10%). Although MIC testing was not performed in the present study, published evidence summarized in our structured literature review indicates that these mutations are frequently associated with MICs near or below the MGIT critical concentration, which may contribute to false-susceptible results. Additional complex mutations and *rpoC* variants (e.g., Lys71Asn) were also observed. Our structured literature review further supported the association of Leu452Pro, Asp435Tyr, and Leu430Pro with discordance across phenotypic methods. These findings underscore the limitations of pDST, particularly MGIT, and highlight the potential value of incorporating WGS as an adjunctive tool in diagnostic workflows, particularly for cases with discordant phenotypic results.

A clinically significant phenotypic discordance rate of 11.7% (31/265) was observed between solid and liquid culture methods, with 17 isolates susceptible by MGIT but resistant by L-J, and 14 exhibiting the reverse. This bidirectional inconsistency mirrors previously reported challenges in TB diagnostics (12, 19, 20). Among 27 retested discordant isolates, more than half (16/27) exhibited results that differed from their initial pDST classification, underscoring the variability and inherent limitations of phenotypic testing. These findings emphasize the importance of confirmatory testing, including molecular diagnostics, in cases of discordant results. Several studies have indicated that the MGIT system may lack sensitivity for detecting low-level resistance, frequently yielding false-susceptible results, especially near the critical concentration threshold or in the presence of heteroresistance (7, 21). Certain *rpoB* mutations such as Leu430Pro, Asp435Tyr, and Leu452Pro are classified as “disputed mutations” that confer low-level RIF resistance (22–24). Published MIC data suggest that these mutations may yield resistance levels near or below the critical threshold, making them difficult to detect via standard MGIT-based pDST, potentially due to slower bacterial growth or reduced adaptability under drug pressure (25, 26). Additionally, variations in growth conditions, inoculum density, and drug diffusion between solid and liquid media may influence the phenotypic expression of resistance (27).

All isolates in this study belonged to the East Asian/Beijing lineage, a globally prevalent genotype strongly associated with MDR-TB (28). Genotypic analysis revealed that all 10 sequenced isolates, each of which was susceptible by MGIT but resistant by L-J, harbored *rpoB* mutations known to confer RIF resistance, thereby reinforcing the utility of molecular diagnostics in resolving phenotypic discrepancies. Among these, Leu452Pro (50%) and Asp435Tyr (10%) were the most frequently identified mutations. These results are consistent with findings from the accompanying structured literature review, which also highlighted Leu430Pro and mutations at codon His445 as commonly associated with phenotypic discordance. In addition to these well-characterized single mutations, several complex double mutations were observed, including Asp435Tyr + Asn437 Asp, Leu430Pro + Asn437 Asp, and Met434Val + Asp435 Gly. Such dual mutations may plausibly be associated with altered resistance expression compared with single mutations, although their isolate-specific MIC distributions were not determined in the present study (19). Notably, some of these mutations, such as Asn437Asp and Met434Val, are rare within the RRDR, and their co-occurrence represents a novel finding. All isolates carried the synonymous *rpoB* mutation Ala1075Ala, a variant strongly associated with the Beijing genotype and consistent with previous reports (29). Additionally, a unique synonymous mutation in *rpoA* (Tyr341Tyr) was detected in isolate A6. Although synonymous mutations do not alter amino acid sequences, molecular tests such as Xpert MTB/RIF may misinterpret these nucleotide changes as resistance-associated, potentially leading to false-positive results (30, 31).

Many of the *rpoB* mutations identified (e.g., Leu452Pro, Asp435Tyr, and Leu430Pro) are widely regarded as “disputed mutations” or “low-level resistance mutations.” Published evidence suggests that these variants may yield MICs at or below the conventional critical concentration of 1.0 µg/mL used in the MGIT system, thereby increasing the risk of false-susceptible results. In response to this diagnostic challenge, the World Health Organization has revised the critical concentration for RIF in MGIT from 1.0 µg/mL to 0.5 µg/mL (32). Nevertheless, some studies have proposed that even lower thresholds, such as 0.25 mg/L (20) or 0.125 mg/L (33), may be necessary to detect the full spectrum of low-level resistance, with the latter achieving up to 98.4% sensitivity and 100% specificity (33). Despite these adjustments, the limited published evidence available to date suggests that phenotypic methods may continue to overlook certain mutations even at reduced critical concentrations (34, 35). However, this conclusion should be interpreted cautiously because only a small number of studies have directly compared different MGIT critical concentrations. This limitation underscores the value of molecular diagnostics, particularly WGS, as a supplementary tool in the detection of drug resistance. WGS enables comprehensive identification of resistance-associated mutations, including rare or non-RRDR variants, thereby informing individualized treatment regimens and mitigating the risk of treatment failure and ongoing transmission of DR-TB. Previous studies have linked such mutations to poor treatment outcomes (36, 37). In this cohort, 5 of 9 patients with available clinical histories had received prior anti-tuberculosis treatment, although no treatment failures were documented. However, due to the small sample size and lack of long-term follow-up, the clinical significance of these mutations could not be fully assessed.

Some isolates (e.g., A1 and A5) also harbored mutations in *rpoC* (Lys71Asn and Glu49Gly) or a synonymous mutation in *rpoA* (Tyr341Tyr). While mutations in *rpoA* and *rpoC* are typically characterized as compensatory adaptations, particularly in association with the high-level resistance mutation *rpoB* Ser450Leu (38, 39), their co-occurrence with low-level resistance mutations, in the absence of Ser450Leu, represents a novel observation. These findings suggest that such compensatory mutations may contribute to the persistence and transmission of low-level RIF-resistant strains, thereby further complicating phenotypic resistance detection. These results highlight the need for comprehensive genotypic analyses that extend beyond RRDR. Given that drug resistance can arise from mutations outside the RRDR and involve mechanisms such as altered cell wall permeability or efflux pump activity, exclusive reliance on conventional DST or molecular methods targeting only RRDR mutations may lead to incomplete or missed diagnoses.

Despite providing valuable insights, this study has several limitations that merit consideration. First, only a subset of discordant isolates underwent WGS, which limits the generalizability of the findings. Second, all sequenced discordant isolates originated from a single center and belonged to the East Asian/Beijing lineage, which may limit extrapolation to other settings and strain backgrounds. Third, MIC testing was not performed, limiting our ability to directly quantify resistance levels for the identified mutation patterns and to assess their relationship to current rifampicin critical concentrations. Fourth, the study did not include phenotypically concordant isolates as a comparator group. Fifth, while *rpoB*, *rpoC*, and *rpoA* mutations were investigated, other potential resistance mechanisms, such as efflux pump activity or metabolic adaptations, were not explored. Finally, clinical outcome data were incomplete, thereby limiting assessment of the real-world impact of these mutations on treatment efficacy and patient outcomes.

In conclusion, more than 10% of MTB isolates in our setting showed reproducible RIF phenotypic discordance between solid (L-J) and liquid (MGIT 960) systems, creating a meaningful risk of misclassification in DR-TB management. Most sequenced discrepancies were associated with specific low-level *rpoB* mutations, particularly Leu452Pro, which have been linked in previous studies to detection challenges in liquid culture-based systems. These findings expose inherent limits of standalone pDST and emphasize the potential value of WGS to adjudicate unclear or conflicting results. Clinical decisions may benefit from integrating both phenotypic and genotypic evidence, especially when resistance is suspected or results disagree. Comprehensive molecular analyses, including WGS, may help reduce the underestimation of resistance and support more informed regimen optimization. Similar mechanisms may underlie discordance for other anti-tuberculosis drugs, and our approach offers a framework for systematically investigating and resolving such discrepancies.

## Supplementary Material

Reviewer comments

## Data Availability

All sequencing data are available in the NCBI SRA database under accession number PRJNA1108515.

## SUPPLEMENTAL MATERIAL

Table S1 spectrum.03261-25-s0001.xlsx The description of phenotype changes before and after retesting.

Table S2 spectrum.03261-25-s0002.xlsx Drug resistance reports generated by the TBProfiler and Mykrobe.

Table S3 spectrum.03261-25-s0003.xlsx Phenotypic discrepancies in RIF susceptibility among MTB isolates with rpoB mutations across different culture systems.

Supplemental material spectrum.03261-25-s0004.docx Search strategy and screening workflow for the literature review.

OPEN PEER REVIEW reviewer-comments.pdf An accounting of the reviewer comments and feedback.

ASM does not own the copyrights to Supplemental Material that may be linked to, or accessed through, an article. The authors have granted ASM a non-exclusive, world-wide license to publish the Supplemental Material files. Please contact the corresponding author directly for reuse.

## References

[1] World Health Organization. 2025. Global tuberculosis report 2025. Geneva World Health Organization

[2] Van Deun Armand, Aung KJM, Bola V, Lebeke R, Hossain MA, de Rijk WB, Rigouts L, Gumusboga A, Torrea G, de Jong BC. 2013. Rifampin drug resistance tests for tuberculosis: challenging the gold standard. J Clin Microbiol 51:2633–2640. doi:10.1128/JCM.00553-13 23761144 PMC3719626

[3] Lempens P, Van Deun A, Aung KJM, Hossain MA, Behruznia M, Decroo T, Rigouts L, de Jong BC, Meehan CJ. 2023. Borderline *rpoB* mutations transmit at the same rate as common *rpoB* mutations in a tuberculosis cohort in Bangladesh. Microb Genom 9:9. doi:10.1099/mgen.0.001109 PMC10569737 37750750

[4] Ocheretina O, Escuyer VE, Mabou M-M, Royal-Mardi G, Collins S, Vilbrun SC, Pape JW, Fitzgerald DW. 2014. Correlation between genotypic and phenotypic testing for resistance to rifampin in *Mycobacterium tuberculosis* clinical isolates in Haiti: investigation of cases with discrepant susceptibility results. PLoS One 9:e90569. doi:10.1371/journal.pone.0090569 24599230 PMC3944071

[5] Van Deun A, Barrera L, Bastian I, Fattorini L, Hoffmann H, Kam KM, Rigouts L, Rüsch-Gerdes S, Wright A. 2009. *Mycobacterium tuberculosis* strains with highly discordant rifampin susceptibility test results. J Clin Microbiol 47:3501–3506. doi:10.1128/JCM.01209-09 19759221 PMC2772627

[6] Van Deun A A, Decroo T, Aung KJM, Hossain MA, Gumusboga M, De Rijk WB, Tahseen S, de Jong BC, Rigouts L. 2021. Mycobacterium tuberculosis borderline rpoB mutations: emerging from the unknown. Eur Respir J:58. doi:10.1183/13993003.00783-2021 33926970

[7] Gonzalo X, Claxton P, Brown T, Montgomery L, Fitzgibbon M, Laurenson I, Drobniewski F. 2017. True rifampicin resistance missed by the MGIT: prevalence of this pheno/genotype in the UK and Ireland after 18 month surveillance. Clin Microbiol Infect 23:260–263. doi:10.1016/j.cmi.2016.11.015 27903459

[8] Tamilzhalagan S, Shanmugam S, Selvaraj A, Suba S, Suganthi C, Moonan PK, Surie D, Sathyanarayanan MK, Gomathi NS, Jayabal L, et al.. 2021. Whole-genome sequencing to identify missed rifampicin and isoniazid resistance among tuberculosis isolates-Chennai, India, 2013-2016. Front Microbiol 12:720436. doi:10.3389/fmicb.2021.720436 34880835 PMC8645853

[9] He G, Zheng Q, Wu J, Wu L, Geng Z, Jiang G, Huang H, Jiang X, Yu X. 2024. Discordant results between Xpert MTB/RIF assay and Bactec MGIT 960 culture system regarding the detection of rifampin-resistant *Mycobacterium tuberculosis* isolates in Wenzhou, China. Microbiol Spectr 12:e0385923. doi:10.1128/spectrum.03859-23 38738892 PMC11237732

[10] Xia H, Song Y, Zheng Y, Wang S, Zhao B, He W, Liu D, Ou X, Zhou Y, Zhao Y. 2022. Detection of *Mycobacterium tuberculosis* rifampicin resistance conferred by borderline *rpoB* mutations: xpert MTB/RIF is superior to phenotypic drug susceptibility testing. Infect Drug Resist 15:1345–1352. doi:10.2147/IDR.S358301 35378895 PMC8976515

[11] Banu S, Rahman SMM, Khan MSR, Ferdous SS, Ahmed S, Gratz J, Stroup S, Pholwat S, Heysell SK, Houpt ER. 2014. Discordance across several methods for drug susceptibility testing of drug-resistant *Mycobacterium tuberculosis* isolates in a single laboratory. J Clin Microbiol 52:156–163. doi:10.1128/JCM.02378-13 24172155 PMC3911413

[12] Shea J, Halse TA, Kohlerschmidt D, Lapierre P, Modestil HA, Kearns CH, Dworkin FF, Rakeman JL, Escuyer V, Musser KA. 2021. Low-level rifampin resistance and *rpoB* mutations in *Mycobacterium tuberculosis*: an analysis of whole-genome sequencing and drug susceptibility test data in New York. J Clin Microbiol 59:59. doi:10.1128/JCM.01885-20 PMC8579749 32999007

[13] Torrea G, Ng KCS, Van Deun A, André E, Kaisergruber J, Ssengooba W, Desmaretz C, Gabriels S, Driesen M, Diels M, et al.. 2019. Variable ability of rapid tests to detect *Mycobacterium tuberculosis* rpoB mutations conferring phenotypically occult rifampicin resistance. Sci Rep 9:11826. doi:10.1038/s41598-019-48401-z 31413308 PMC6694172

[14] Miotto P, Cabibbe AM, Borroni E, Degano M, Cirillo DM. 2018. Role of disputed mutations in the *rpoB* gene in interpretation of automated liquid mgit culture results for rifampin susceptibility testing of *Mycobacterium tuberculosis**.* J Clin Microbiol 56:01599–17. doi:10.1128/JCM.01599-17 PMC5925711 29540456

[15] Jose Vadakunnel M, Nehru VJ, Brammacharry U, Ramachandra V, Palavesam S, Muthukumar A, Mani BR, R SS, Pradhabane G, Vn AD, et al.. 2025. Impact of rpoB gene mutations and rifampicin-resistance levels on treatment outcomes in rifampicin-resistant tuberculosis. BMC Infect Dis 25:284. doi:10.1186/s12879-025-10655-6 40016696 PMC11866845

[16] Abueg LAL, Afgan E, Allart O, Awan AH, Bacon WA, Baker D, Bassetti M, Batut B, Bernt M, Blankenberg D, et al.. 2024. The galaxy platform for accessible, reproducible, and collaborative data analyses: 2024 update. Nucleic Acids Res 52:W83–W94. doi:10.1093/nar/gkae410 38769056 PMC11223835

[17] Phelan JE, O’Sullivan DM, Machado D, Ramos J, Oppong YEA, Campino S, O’Grady J, McNerney R, Hibberd ML, Viveiros M, et al.. 2019. Integrating informatics tools and portable sequencing technology for rapid detection of resistance to anti-tuberculous drugs. Genome Med 11:41. doi:10.1186/s13073-019-0650-x 31234910 PMC6591855

[18] Bradley P, Gordon NC, Walker TM, Dunn L, Heys S, Huang B, Earle S, Pankhurst LJ, Anson L, de Cesare M, et al.. 2015. Rapid antibiotic-resistance predictions from genome sequence data for *Staphylococcus aureus* and *Mycobacterium tuberculosis**.* Nat Commun 6:10063. doi:10.1038/ncomms10063 26686880 PMC4703848

[19] Qadir M, Faryal R, Khan MT, Khan SA, Zhang S, Li W, Wei DQ, Tahseen S, McHugh TD. 2024. Phenotype versus genotype discordant rifampicin susceptibility testing in tuberculosis: implications for a diagnostic accuracy. Microbiol Spectr 12:e0163123. doi:10.1128/spectrum.01631-23 37982632 PMC10783056

[20] Rigouts L, Gumusboga M, de Rijk WB, Nduwamahoro E, Uwizeye C, de Jong B, Van Deun A. 2013. Rifampin resistance missed in automated liquid culture system for *Mycobacterium tuberculosis* isolates with specific *rpoB* mutations. J Clin Microbiol 51:2641–2645. doi:10.1128/JCM.02741-12 23761146 PMC3719602

[21] Issues in *Mycobacterium tuberculosis* complex drug susceptibility testing: rifampin. 2022. Association of Public Health Laboratories. Available from: https://www.aphl.org/aboutAPHL/publications/Documents/ID-2022-MTBC-DST-Rifampin.pdf

[22] Abanda NN, Djieugoué JY, Khadka VS, Pefura-Yone EW, Mbacham WF, Vernet G, Penlap VM, Deng Y, Eyangoh SI, Taylor DW, et al.. 2018. Absence of hybridization with the wild-type and mutant rpoB probes in the genotype MTBDRplus assay detects “disputed” rifampicin mutations. Clin Microbiol Infect 24:781. doi:10.1016/j.cmi.2017.11.021 PMC5988914 29217277

[23] Miotto P, Cabibbe AM, Borroni E, Degano M, Cirillo DM. 2018. Role of disputed mutations in the *rpoB* gene in interpretation of automated liquid mgit culture results for rifampin susceptibility testing of *Mycobacterium tuberculosis*. J Clin Microbiol 56:56. doi:10.1128/JCM.01599-17 PMC5925711 29540456

[24] Van Deun A, Aung KJM, Hossain A, de Rijk P, Gumusboga M, Rigouts L, de Jong BC. 2015. Disputed *rpoB* mutations can frequently cause important rifampicin resistance among new tuberculosis patients. Int J Tuberc Lung Dis 19:185–190. doi:10.5588/ijtld.14.0651 25574917

[25] Leehan JD, Nicholson WL. 2022. Environmental dependence of competitive fitness in rifampin-resistant *rpoB* mutants of *Bacillus subtilis*. Appl Environ Microbiol 88:e0242221. doi:10.1128/aem.02422-21 35258334 PMC8904044

[26] Mariam DH, Mengistu Y, Hoffner SE, Andersson DI. 2004. Effect of rpoB mutations conferring rifampin resistance on fitness of *Mycobacterium tuberculosis*. Antimicrob Agents Chemother 48:1289–1294. doi:10.1128/AAC.48.4.1289-1294.2004 15047531 PMC375340

[27] Balouiri M, Sadiki M, Ibnsouda SK. 2016. Methods for *in vitro* evaluating antimicrobial activity: a review. J Pharm Anal 6:71–79. doi:10.1016/j.jpha.2015.11.005 29403965 PMC5762448

[28] Parwati I, van Crevel R, van Soolingen D. 2010. Possible underlying mechanisms for successful emergence of the *Mycobacterium tuberculosis* Beijing genotype strains. Lancet Infect Dis 10:103–111. doi:10.1016/S1473-3099(09)70330-5 20113979

[29] Wan L, Liu H, Li M, Jiang Y, Zhao X, Liu Z, Wan K, Li G, Guan C-X. 2020. Genomic Analysis identifies mutations concerning drug-resistance and beijing genotype in multidrug-resistant *Mycobacterium tuberculosis* isolated from China. Front Microbiol 11:1444. doi:10.3389/fmicb.2020.01444 32760357 PMC7373740

[30] Alonso M, Palacios JJ, Herranz M, Penedo A, Menéndez A, Bouza E, García de Viedma D. 2011. Isolation of *Mycobacterium tuberculosis* strains with a silent mutation in rpoB leading to potential misassignment of resistance category. J Clin Microbiol 49:2688–2690. doi:10.1128/JCM.00659-11 21562104 PMC3147855

[31] Mathys V, van de Vyvere M, de Droogh E, Soetaert K, Groenen G. 2014. False-positive rifampicin resistance on Xpert MTB/RIF caused by a silent mutation in the rpoB gene. Int J Tuberc Lung Dis 18:1255–1257. doi:10.5588/ijtld.14.0297 25216843

[32] World Health Organization. 2021. Technical report on critical concentrations for drug susceptibility testing of isoniazid and the rifamycins (rifampicin, rifabutin and rifapentine). Geneva World Health Organization

[33] Wang W, Liu R, Yao C, Huo F, Shang Y, Zhang X, Wang Y, Xue Z, Ma L, Pang Y. 2022. Reevaluating rifampicin breakpoint concentrations for *Mycobacterium tuberculosis* isolates with disputed *rpoB* mutations and discordant susceptibility phenotypes. Microbiol Spectr 10:e0208721. doi:10.1128/spectrum.02087-21 35107324 PMC8809345

[34] Yu H-J, Kim TY, Kim G, Shim HJ, Kang OK, Kim S, Kim C-K, Jhun BW, Lee NY, Huh HJ, et al.. 2023. Performance evaluation of the BACTEC MGIT 960 system for rifampin drug-susceptibility testing of *Mycobacterium tuberculosis* using the current WHO critical concentration. J Clin Microbiol 61:e0108622. doi:10.1128/jcm.01086-22 36602360 PMC9879093

[35] Rupasinghe P, Ashraf A, Barreda N, Parveen S, Zubair M, Calderon R, Asif S, Hirani N, Chingisova L, Bulane A, et al.. 2024. Reduced critical concentration might not have improved MGIT-based DST’s sensitivity to rifampicin. Antimicrob Agents Chemother 68:e0170123. doi:10.1128/aac.01701-23 38534101 PMC11064607

[36] Shah N, Lin G, Barry P, Schecter G, Desmond E. 2015. Clinical Impact of rpob mutations conferring discordant phenotypic-genotypic rifampin resistance in *Mycobacterium tuberculosis*. Open Forum Infect Dis 2:571. doi:10.1093/ofid/ofv133.445

[37] Lin W-H, Lee W-T, Tsai H-Y, Jou R. 2021. Disputed *rpoB* mutations in *Mycobacterium tuberculosis* and tuberculosis treatment outcomes. Antimicrob Agents Chemother 65:e0157320. doi:10.1128/AAC.01573-20 33846134 PMC8218645

[38] de Vos M, Müller B, Borrell S, Black PA, van Helden PD, Warren RM, Gagneux S, Victor TC. 2013. Putative compensatory mutations in the rpoC gene of rifampin-resistant *Mycobacterium tuberculosis* are associated with ongoing transmission. Antimicrob Agents Chemother 57:827–832. doi:10.1128/AAC.01541-12 23208709 PMC3553702

[39] Li Q, Jiao W, Yin Q, Xu F, Li J, Sun L, Xiao J, Li Y, Mokrousov I, Huang H, et al.. 2016. Compensatory mutations of rifampin resistance are associated with transmission of multidrug-resistant *Mycobacterium tuberculosis* beijing genotype strains in china. Antimicrob Agents Chemother 60:2807–2812. doi:10.1128/AAC.02358-15 26902762 PMC4862491

