# Supplementary material for: Phenotypic discordance in rifampicin resistance detection among Mycobacterium tuberculosis isolates from China: insights from whole-genome sequencing and a structured literature review
Source: Microbiol Spectr. 2026 Jul 6;14(8):e03261-25. doi: 10.1128/spectrum.03261-25 (PMC13435698; doi:10.1128/spectrum.03261-25)
Supplement: Supplementary file 1 [file reviewer-comments.pdf]

AMERICAN SOCIETY FOR MICROBIOLOGY | Microbiology Spectrum

Phenotypic discordance in rifampicin resistance detection among *Mycobacterium tuberculosis* isolates from China: insights from whole genome sequencing and a structured literature review

Ya-Li Chen, Cui-Ping Guan, Yunfeng Deng, Mao-Shui Wang, and Zhongfa Zhang

Corresponding Author(s): Mao-Shui Wang, Shandong Public Health Clinical Center

Review Timeline:

Submission Date:	October 9, 2025
Editorial Decision:	April 15, 2026
Revision Received:	May 27, 2026
Accepted:	June 1, 2026

Editor: Michael Whitfield

Reviewer(s): The reviewers have opted to remain anonymous.

Transaction Report:

(Note: With the exception of the correction of typographical or spelling errors that could be a source of ambiguity, letters and reports are not edited. The original formatting of letters and referee reports may not be reflected in this compilation.)

DOI: https://doi.org/10.1128/spectrum.03261-25

Re: Spectrum03261-25 (Phenotypic discordance in rifampicin resistance detection among Mycobacterium tuberculosis isolates from China: insights from whole genome sequencing and systematic review)

Dear Dr. Mao-Shui Wang:

Thank you for the privilege of reviewing your work. Below you will find my comments, instructions from the Spectrum editorial office, and the reviewer comments.

Please return the manuscript within 60 days; if you cannot complete the modification within this time period, please contact me. If you do not wish to modify the manuscript and prefer to submit it to another journal, notify me immediately so that the manuscript may be formally withdrawn from consideration by Spectrum.

Revision Guidelines
To submit your modified manuscript, log into the submission site at https://spectrum.msubmit.net/cgi-bin/main.plex. Go to Author Tasks and click the appropriate manuscript title to begin. The information you entered when you first submitted the paper will be displayed; update this as necessary. Note the following requirements:

• Upload point-by-point responses to the issues raised by the reviewers in a file named "Response to Reviewers," NOT in your cover letter.
• Upload a compare copy of the manuscript (without figures) as a "Marked-Up Manuscript" file.
• Upload a clean .DOC/.DOCX version of the revised manuscript and remove the previous version.
• Each figure must be uploaded as a separate, editable, high-resolution file (TIFF or EPS preferred), and any multipanel figures must be assembled into one file.
• Any supplemental material intended for posting by ASM should be uploaded with their legends separate from the main manuscript. You can combine all supplemental material into one file (preferred) or split it into a maximum of 10 files with all associated legends included.

For complete guidelines on revision requirements, see our Submission and Review Process webpage. Submission of a paper that does not conform to guidelines may delay acceptance of your manuscript.

Data availability: ASM policy requires that data be available to the public upon online posting of the article, so please verify all links to sequence records, if present, and make sure that each number retrieves the full record of the data. If a new accession number is not linked or a link is broken, provide Spectrum production staff with the correct URL for the record. If the accession numbers for new data are not publicly accessible before the expected online posting of the article, publication may be delayed; please contact production staff (Spectrum@asmusa.org) immediately with the expected release date.

Publication Fees: For information on publication fees and which article types are subject to charges, visit ourwebsite. If your manuscript is accepted for publication and any fees apply, you will be contacted separately about payment during the production process; please follow the instructions in that e-mail. Arrangements for payment must be made before your article is published.

ASM Membership: Corresponding authors may join or renew ASM membership to obtain discounts on publication fees. Need to upgrade your membership level? Please contact Customer Service at Service@asmusa.org.

The ASM Journals program strives for constant improvement in our submission and publication process. Please tell us how we can improve your experience by taking this quick Author Survey.

Thank you for submitting your paper to Spectrum.

Sincerely,
Michael Whitfield
Editor
Microbiology Spectrum

Editors comments:
1)While we are willing to consider a revised version of this paper at Spectrum, it would be in your best interest to improve the writing. I recommend that you ask a colleague of yours who is a native English speaker to read and provide you some feedback on the writing. You are also welcome to use one of the services here: https://journals.asm.org/writing-your-paper#language-editing-services
2) Please include a data availability statement regarding the WGS conducted along with the public repository identifier as per the journals requirements.

Reviewer #1 (Comments for the Author):

After reviewing the manuscript entitled "Phenotypic discordance in rifampicin resistance detection among Mycobacterium tuberculosis isolates from China: insights from whole genome sequencing and systematic review," I find the topic relevant . Disputed mutations in the rpoB gene remain a major challenge for rifampicin resistance detection using phenotypic DST methods, particularly with the MGIT 960 system. However, the study presents important gaps, notably concerning the rifampicin critical concentration (cc) applied and the absence of MIC testing to explain missed detections by MGIT 960

Major Comments
Comment 1
Lines 142-153: In this section of the Results, it is difficult to clearly follow the transitions from susceptible to resistant results (and vice versa), as well as to identify strains that retained the same phenotype after retesting. Presenting these data in a table summarizing pDST results before and after retesting would greatly improve clarity.
Based on my understanding, only strains that remained discordant after retesting were subjected to whole-genome sequencing. It would also be informative to sequence strains that were initially discordant between LJ and MGIT 960 but became concordant after retesting, as these isolates may also harbor disputed rpoB mutations. What was the rationale for excluding these strains from sequencing? Were they at least tested using other molecular methods (e.g., Xpert MTB/RIF or line probe assays) to confirm the absence of mutations at least in the rpoB hotspot region?"
Comment 2
The absence of MIC testing represents a major limitation of the study. At a minimum, testing the strains at both critical concentrations (1.0 µg/mL and 0.5 µg/mL) would have provided valuable insight. MIC distributions are influenced by multiple factors, including the genetic background of the strains. Therefore, determining MIC values for each mutation particularly in East Asian Beijing lineage strains would help to more accurately assess the new rifampicin critical concentration recommended by the WHO.
Comment 3
"Adjusting the RIF critical concentration in MGIT from 1.0 µg/mL to 0.5 µg/mL had only a minor impact on the discordance rates: Asp435Tyr (35.29% vs. 35.67%), Leu452Pro (27.45% vs. 19.88%), Leu430Pro (11.76% vs. 10.53%), and His445 mutations (19.61% vs. 20.47%)."
Could the authors clarify how many studies this conclusion is based on? Did all included studies evaluate the same strains using both critical concentrations (1.0 µg/mL and 0.5 µg/mL)? Based on Table S3, only two studies appear to have tested strains using both critical concentrations: Rigouts et al., 2013 and Hui-Jin Yu et al., 2023. This limited number of studies may not be sufficient to draw definitive conclusions. Additional studies involving diverse geographical settings and a wider range of M. tuberculosis lineages are needed to more robustly evaluate the new critical concentration or to support the proposal of an alternative cc.
For Table S3, if possible, please consider adding a column describing the most frequent lineages reported in each study, where such data are available.

Minor Comments
• Update epidemiological statistics using the 2025 Global TB Report.
• Combine mutations identified by TB-Profiler and Mykrobe into a single table for clarity and to could compare mutations identified by the two tools.
• Specify manufacturers for all reagents and instruments used (e.g., MGIT, Qubit).

Reviewer #2 (Comments for the Author):

Comment 1: The authors state that WGS resolved all discrepant cases, however this be my overstated as only a fraction of all discrepant cases were sequenced. In addition, the authors do not have any comparison with non-discrepant cases. Suggest that the authors rephrase the conclusions to be more appropriate.
Comment 2: In the discussion the authors note that discordance may arise from MICs near critical concentrations, yet the authors have conducted no MIC testing on their discrepant cases. Why was MIC testing not conducted? Suggest the authors to include MIC data if available or soften their claims about resistance levels.
Comment 3: The authors state that a systematic review was conducted, however the methods and results presented do not met the threshold for a systematic review. There are no details of PRISMA adherence, no information on study selection criteria or critical assessment (risk of bias). This raises concerns on reproducibility and validity of the work.
Comment 4: The authors advocate for routine integration of WGS and clinical decision-making changes, which are strong claims to be made. While there is lots of evidence to support WGS integration into routine practice and some countries/regions are doing this already, the authors need to rephrase sections to more closely align with their own findings. Factors such as data is from a single center and all Beijing lineage isolates present should be noted in the limitations.

Minor comments:
Comment 5: rifampicin and rifampin are used interchangeable. Please be consistent.
Comment 6: UI is not defined in the introduction.
Comment 7: Reference 12 and 21 appear to be duplicate references.
Comment 8: line 68 "pDST, which based on growth ..." should be "which is based on the growth ..."
Comment 9: line 195 "265 MTB isolates which tested for RIF" should be "265 MTB isolates which were tested for RIF".
Comment 10: There are no definitions for the outcomes achieved.

After reviewing the manuscript entitled "Phenotypic discordance in rifampicin resistance detection among *Mycobacterium tuberculosis* isolates from China: insights from whole genome sequencing and systematic review," I find the topic relevant. Disputed mutations in the *rpoB* gene remain a major challenge for rifampicin resistance detection using phenotypic DST methods, particularly with the MGIT 960 system. However, the study presents important gaps, notably concerning the rifampicin critical concentration applied and the absence of MIC testing to explain missed detections by MGIT 960

Major Comments

Comment 1

Lines 142–153: In this section of the Results, it is difficult to clearly follow the transitions from susceptible to resistant results (and vice versa), as well as to identify strains that retained the same phenotype after retesting. Presenting these data in a table summarizing pDST results before and after retesting would greatly improve clarity.

Based on my understanding, only strains that remained discordant after retesting were subjected to whole-genome sequencing. It would also be informative to sequence strains that were initially discordant between LJ and MGIT 960 but became concordant after retesting, as these isolates may also harbor disputed *rpoB* mutations. What was the rationale for excluding these strains from sequencing? Were they at least tested using other molecular methods (e.g., Xpert MTB/RIF or line probe assays) to confirm the absence of mutations at least in the *rpoB* hotspot region?"

Comment 2

The absence of MIC testing represents a major limitation of the study. At a minimum, testing the strains at both critical concentrations (1.0 µg/mL and 0.5 µg/mL) would have provided valuable insight. MIC distributions are influenced by multiple factors, including the genetic background of the strains. Therefore, determining MIC values for each mutation particularly in East Asian Beijing lineage strains would help to more accurately assess the new rifampicin critical concentration recommended by the WHO.

Comment 3

"Adjusting the RIF critical concentration in MGIT from 1.0 µg/mL to 0.5 µg/mL had only a minor impact on the discordance rates: Asp435Tyr (35.29% vs. 35.67%), Leu452Pro (27.45% vs. 19.88%), Leu430Pro (11.76% vs. 10.53%), and His445 mutations (19.61% vs. 20.47%)."

Could the authors clarify how many studies this conclusion is based on? Did all included studies evaluate the same strains using both critical concentrations (1.0 µg/mL and 0.5

µg/mL)? Based on Table S3, only two studies appear to have tested strains using both critical concentrations: **Rigouts et al., 2013** and **Hui-Jin Yu et al., 2023**. This limited number of studies may not be sufficient to draw definitive conclusions. Additional studies involving diverse geographical settings and a wider range of *M. tuberculosis* lineages are needed to more robustly evaluate the new critical concentration or to support the proposal of an alternative threshold.

For Table S3, if possible, please consider adding a column describing the most frequent lineages reported in each study, where such data are available.

Minor Comments

- Update epidemiological statistics using the 2025 Global TB Report.
- Combine mutations identified by TB-Profiler and Mykrobe into a single table for clarity and to could compare mutations identified by the two tools.
- Specify manufacturers for all reagents and instruments used (e.g., MGIT, Qubit).

Dear Dr. N. Esther Babady,

We sincerely appreciate the time and effort that you and the reviewers have devoted to evaluating our manuscript, entitled "Phenotypic discordance in rifampicin resistance detection among *Mycobacterium tuberculosis* isolates from China: insights from whole genome sequencing and a structured literature review" (Manuscript ID: Spectrum03261-25).

We are grateful for the valuable and constructive comments provided, which have helped us improve the quality and clarity of our work substantially. We have carefully considered all of the comments and suggestions and have revised the manuscript accordingly.

In this revised submission, we have included the following materials:

1. A detailed "Response to Reviewers" document addressing each comment point by point.
2. A marked-up version of the manuscript showing all revisions made in response to the reviewers' comments.
3. A clean version of the revised manuscript without tracked changes.

We hope that the revised manuscript meets the standards of Microbiology Spectrum and satisfactorily addresses the concerns raised by the reviewers and the editor. We would be pleased to provide any additional information or make further revisions if necessary.

Thank you again for your consideration of our work. We look forward to hearing from you.

Yours sincerely,

Mao-Shui Wang
Public Health Clinical Center Affiliated to Shandong University

Editors comments:

1) While we are willing to consider a revised version of this paper at Spectrum, it would be in your best interest to improve the writing. I recommend that you ask a colleague of yours who is a native English speaker to read and provide you some feedback on the writing. You are also welcome to use one of the services here: https://journals.asm.org/writing-your-paper#language-editing-services

Responses:

We thank the editor for this valuable suggestion. The manuscript has been comprehensively revised to improve language clarity, grammar, readability, and overall flow. We carefully edited the text throughout the manuscript, simplified sentence structures where appropriate, and standardized terminology to enhance clarity and consistency. We believe that these revisions have substantially improved the readability of the manuscript.

2) Please include a data availability statement regarding the WGS conducted along with the public repository identifier as per the journals requirements.

Responses:

We appreciate this important suggestion. A data availability statement has now been added to the manuscript. The whole genome sequencing (WGS) data generated in this study have been deposited in the NCBI Sequence Read Archive under accession number PRJNA1108515. This information has been clearly stated in the Methods section.

Reviewer #1 (Comments for the Author):

After reviewing the manuscript entitled "Phenotypic discordance in rifampicin resistance detection among Mycobacterium tuberculosis isolates from China: insights from whole genome sequencing and systematic review," I find the topic relevant. Disputed mutations in the rpoB gene remain a major challenge for rifampicin resistance detection using phenotypic DST methods, particularly with the MGIT 960 system. However, the study presents important gaps, notably concerning the rifampicin critical concentration (cc) applied and the absence of MIC testing to explain missed detections by MGIT 960.

Major Comments

Comment 1

Lines 142-153: In this section of the Results, it is difficult to clearly follow the transitions from susceptible to resistant results (and vice versa), as well as to identify strains that retained the same phenotype after retesting. Presenting these data in a table summarizing pDST results before and after retesting would greatly improve clarity.

Responses:

We thank the reviewer for this important suggestion. We agree that the original presentation of the retesting results was not sufficiently clear. In the revised manuscript, we have reorganized this section and added a summary table (Supplementary Table S1) to clearly present the initial and repeat phenotypic drug susceptibility testing (pDST) results for the discordant isolates, including the direction of phenotype changes and the isolates whose results remained unchanged upon retesting.

Based on my understanding, only strains that remained discordant after retesting were subjected to whole-genome sequencing. It would also be informative to sequence strains that were initially discordant between LJ and MGIT 960 but became concordant after retesting, as these isolates may also harbor disputed rpoB mutations. What was the rationale for excluding these strains from sequencing? Were they at least tested using other molecular methods (e.g., Xpert MTB/RIF or line probe assays) to confirm the absence of mutations at least in the rpoB hotspot region?"

Responses:

We would also like to clarify our approach to isolate selection for whole-genome sequencing (WGS). Our selection was not based specifically on whether isolates remained discordant between the L-J and MGIT methods after repeat testing. Rather, isolates were selected for WGS when the repeat pDST results were concordant with their respective initial pDST results, as this was considered to indicate a more stable phenotypic pattern and to reduce the likelihood that the observed results were attributable to technical fluctuation. In practice, the isolates meeting this criterion happened to be those that remained discordant between the two phenotypic methods upon retesting. By contrast, isolates in which either the L-J or MGIT result changed on repeat testing were not included in the WGS analysis.

The rationale for this approach was twofold. First, changes in pDST classification on repeat testing may reflect methodological variability around the critical concentration rather than a stable resistance phenotype. Second, such unstable or transiently discordant results may also arise from more complex biological mechanisms, including heteroresistance, which were beyond the scope of the present study. We fully agree that these isolates remain scientifically important, and

investigating the mechanisms underlying such phenotypic instability is a key focus of our ongoing work. Related studies are currently underway.

We also acknowledge the reviewer's point that isolates showing initial discordance but altered results on retesting may still harbor disputed or borderline resistance-associated mutations. However, to strengthen genotype–phenotype interpretation in the present study, we prioritized isolates with reproducible pDST patterns for WGS analysis. In addition, these isolates were not further evaluated by other molecular assays such as Xpert MTB/RIF or line probe assays as part of this study. We have now clarified this point in the revised manuscript and acknowledged it as a limitation.

Comment 2

The absence of MIC testing represents a major limitation of the study. At a minimum, testing the strains at both critical concentrations (1.0 μg/mL and 0.5 μg/mL) would have provided valuable insight. MIC distributions are influenced by multiple factors, including the genetic background of the strains. Therefore, determining MIC values for each mutation particularly in East Asian Beijing lineage strains would help to more accurately assess the new rifampicin critical concentration recommended by the WHO.

Responses:

We thank the reviewer for these important comments and fully agree that the absence of isolate-specific MIC testing is a major limitation of the present study. Direct MIC determination for our discrepant isolates would have provided stronger evidence for interpreting the phenotypic effects of the identified mutations and for assessing their relationship to current rifampicin critical concentrations.

MIC testing was not performed because this study was designed as a retrospective analysis of archived clinical isolates for which routine phenotypic drug susceptibility testing (pDST) on L-J medium and MGIT 960 had already been completed. At the time of study design and sample processing, MIC determination was not incorporated into the laboratory workflow. In addition, not all isolates remained available in sufficient condition or quantity for expanded downstream phenotypic testing.

At the same time, we would like to clarify that our interpretation was not based solely on inference from genotype in isolation. In addition to the WGS analysis of our own isolates, we conducted a structured literature review specifically focused on phenotypic discordance in rifampicin susceptibility across solid and liquid culture systems in MTB isolates with rpoB mutations. Importantly, the principal mutations identified in our sequenced isolates—especially Leu452Pro, Asp435Tyr, and Leu430Pro—have been repeatedly reported in previous studies as borderline/disputed rifampicin-resistance mutations associated with MGIT false-susceptible results and discordance between solid media and MGIT. As summarized in Table S3, these mutations have been observed in studies using MGIT at 1.0 μg/mL, and for some mutations also at 0.5 μg/mL, where discordance still existed.

Specifically, Leu452Pro, which was the most common mutation in our cohort, has been consistently associated in multiple studies with low-level rifampicin resistance and failure of MGIT to detect resistance at the conventional critical concentration, with some reports indicating persistent discordance even after lowering the MGIT concentration to 0.5 μg/mL.

Similarly, Asp435Tyr has been one of the most frequently reported disputed mutations across studies and has shown recurrent discordance between solid media and MGIT, including reports at both 1.0 μg/mL and 0.5 μg/mL. Leu430Pro has likewise been repeatedly documented as a mutation associated with low-level resistance and MGIT underdetection. Therefore, although we did not perform MIC testing on our own isolates, the interpretation that these mutations are linked to low-level or borderline rifampicin resistance is supported by previously published evidence summarized in our review.

For the more complex mutation patterns identified in our study, such as Asp435Tyr + Asn437Asp, Leu430Pro + Asn437Asp, and Met434Val + Asp435Gly, we agree that the evidence is less direct and should be interpreted more cautiously. While these compound RRDR mutations may plausibly contribute to phenotypic instability or altered resistance expression, our study does not directly establish their MIC distributions, and further work will be required to define their phenotypic impact.

Following the reviewers' suggestions, we have now explicitly acknowledged the lack of MIC data as a major limitation in the revised manuscript and have moderated the relevant statements in the Discussion. Accordingly, these interpretations are now framed more cautiously and are based on previously published evidence for the identified rpoB mutations, rather than being presented as findings directly demonstrated in our own isolates. In future work, we will aim to incorporate MIC testing, including gradient-based approaches where feasible. Such studies should assess MICs at 1.0 μg/mL and 0.5 μg/mL, and ideally across a wider range of concentrations, to more precisely define mutation-specific resistance patterns, particularly in Beijing-lineage strains.

Comment 3

"Adjusting the RIF critical concentration in MGIT from 1.0 µg/mL to 0.5 µg/mL had only a minor impact on the discordance rates: Asp435Tyr (35.29% vs. 35.67%), Leu452Pro (27.45% vs. 19.88%), Leu430Pro (11.76% vs. 10.53%), and His445 mutations (19.61% vs. 20.47%)."

Could the authors clarify how many studies this conclusion is based on? Did all included studies evaluate the same strains using both critical concentrations (1.0 µg/mL and 0.5 µg/mL)? Based on Table S3, only two studies appear to have tested strains using both critical concentrations: Rigouts et al., 2013 and Hui-Jin Yu et al., 2023. This limited number of studies may not be sufficient to draw definitive conclusions. Additional studies involving diverse geographical settings and a wider range of M. tuberculosis lineages are needed to more robustly evaluate the new critical concentration or to support the proposal of an alternative cc.

For Table S3, if possible, please consider adding a column describing the most frequent lineages reported in each study, where such data are available.

Responses:

We thank the reviewer for this careful and valuable suggestion. We agree that the evidence base for comparing discordance at MGIT critical concentrations of 1.0 μg/mL and 0.5 μg/mL is limited, and that our original wording was too definitive. In the revised manuscript, we have therefore moderated the relevant statement accordingly.

Specifically, only a small number of published studies directly reported discordance data at both MGIT critical concentrations, and not all studies assessed the same isolates under both conditions. Therefore, the currently available evidence is insufficient to support a firm conclusion regarding

the overall impact of lowering the MGIT rifampicin critical concentration from 1.0 μg/mL to 0.5 μg/mL. We have revised the manuscript to state more cautiously that lowering the critical concentration may not fully resolve discordance for some rpoB mutations, but that the available evidence remains limited and further studies are needed.

We also appreciate the reviewer's suggestion to include lineage information in Table S3. In the revised supplementary table, we have added a new column summarizing the reported lineage distribution or genotype background where such information was available from the original publications. Only four of the included studies provided lineage-level information that could be directly extracted for the discordant isolates. Among these studies, discordant rifampicin susceptibility was observed across multiple genetic backgrounds, including Lineage 2 (Beijing), Lineage 3 (Central Asian), and Lineage 4 (Euro-American) strains. These findings suggest that the association between key rpoB mutations and phenotypic discordance is not restricted to a single lineage background. However, because lineage information was unavailable for most included studies, we agree that the current evidence remains insufficient to formally assess the effect of genetic background on MIC distributions or on the likelihood of discordant phenotypic results. We have now clarified this point in the revised manuscript.

Minor Comments

• Update epidemiological statistics using the 2025 Global TB Report.

Responses:

Thank you for this suggestion. We have updated the epidemiological background in the Introduction using the most recent Global Tuberculosis Report 2025, where appropriate in the revised manuscript.

• Combine mutations identified by TB-Profiler and Mykrobe into a single table for clarity and to could compare mutations identified by the two tools.

Responses:

We thank the reviewer for this helpful suggestion. In the revised manuscript, we have combined the mutation results identified by TB-Profiler and Mykrobe into a single integrated table (revised as Table S2). This unified table presents mutations detected by both tools side-by-side for each isolate, allowing for a direct comparison of their outputs. This revision improves clarity, facilitates interpretation of concordance between the two pipelines, and enhances the overall readability of the results. We observed a high level of concordance between the two tools.

• Specify manufacturers for all reagents and instruments used (e.g., MGIT, Qubit).

Responses:

Thank you for pointing this out. We have carefully reviewed the Materials and Methods section and added or standardized manufacturer information for the major reagents and instruments used in this study.

Reviewer #2 (Comments for the Author):

Comment 1: The authors state that WGS resolved all discrepant cases, however this be my overstated as only a fraction of all discrepant cases were sequenced. In addition, the authors do not have any comparison with non-discrepant cases. Suggest that the authors rephrase the conclusions to be more appropriate.

Responses:

We thank the reviewer for this important comment and agree that our original wording was too strong. Only a subset of discordant isolates was included in the WGS analysis, and these were selected based on the reproducibility of their phenotypic drug susceptibility testing (pDST) results upon repeat testing. In addition, we did not include non-discordant isolates as a comparator group. Therefore, it is not appropriate to generalize our sequencing findings to all discrepant isolates.

In the revised manuscript, we have carefully moderated the relevant statements in the Abstract, Discussion, and Conclusion. Specifically, we now state that WGS identified resistance-associated mutations in all sequenced discrepant isolates, rather than implying that WGS resolved all discrepant cases overall. We have also explicitly acknowledged in the limitations section that only a subset of discordant isolates underwent WGS and that no comparison with phenotypically concordant isolates was performed.

Comment 2: In the discussion the authors note that discordance may arise from MICs near critical concentrations, yet the authors have conducted no MIC testing on their discrepant cases. Why was MIC testing not conducted? Suggest the authors to include MIC data if available or soften their claims about resistance levels.

Responses:

We thank the reviewer for this important comment and fully agree that the absence of isolate-specific MIC testing is a major limitation of the present study. Direct MIC determination for our discrepant isolates would have provided stronger evidence for interpreting the phenotypic effects of the identified mutations and for assessing their relationship to current rifampicin critical concentrations.

MIC testing was not performed because this study was designed as a retrospective analysis of archived clinical isolates for which routine phenotypic drug susceptibility testing (pDST) on L-J medium and MGIT 960 had already been completed. At the time of study design and sample processing, MIC determination was not incorporated into the laboratory workflow. In addition, not all isolates remained available in sufficient condition or quantity for expanded downstream phenotypic testing.

At the same time, we would like to clarify that our interpretation was not based solely on inference from genotype in isolation. In addition to the WGS analysis of our own isolates, we conducted a structured literature review specifically focused on phenotypic discordance in rifampicin susceptibility across solid and liquid culture systems in MTB isolates with rpoB mutations. The principal mutations identified in our sequenced isolates—especially Leu452Pro, Asp435Tyr, and Leu430Pro—have been repeatedly reported in previous studies as borderline/disputed rifampicin-resistance mutations associated with MGIT false-susceptible results and discordance between solid media and MGIT. As summarized in Table S3, these mutations have been observed

in studies using MGIT at 1.0 μg/mL, and for some mutations also at 0.5 μg/mL, where discordance still persisted.

Following the reviewer's suggestion, we have explicitly acknowledged the lack of MIC data as a major limitation in the revised manuscript and have moderated the relevant statements in the Discussion. Accordingly, these interpretations are now framed more cautiously and are based on previously published evidence for the identified rpoB mutations, rather than being presented as findings directly demonstrated in our own isolates.

Comment 3: The authors state that a systematic review was conducted, however the methods and results presented do not met the threshold for a systematic review. There are no details of PRISMA adherence, no information on study selection criteria or critical assessment (risk of bias). This raises concerns on reproducibility and validity of the work.

Responses:

We thank the reviewer for this important observation and agree that the literature review component in our original submission was inappropriately described as a structured literature review. We did not undertake PRISMA-guided reporting, formal study selection documentation, or risk-of-bias assessment, and therefore the use of that term was not sufficiently justified.

To address this concern, we have revised the manuscript throughout and replaced "systematic review" with "structured literature review". We have also revised the corresponding text in the title, abstract, Methods, and Discussion to ensure that the scope and methodological rigor of this component are represented more accurately. The purpose of this literature review was to provide a structured summary of published reports on rpoB mutations associated with phenotypic rifampicin discordance and to contextualize our findings, rather than to serve as a formal structured literature review. We have retained the database sources, search timeframe, and detailed search strategy in Files S1, and we now explicitly acknowledge the absence of formal structured literature review methodology as a limitation of the study.

Comment 4: The authors advocate for routine integration of WGS and clinical decision-making changes, which are strong claims to be made. While there is lots of evidence to support WGS integration into routine practice and some countries/regions are doing this already, the authors need to rephrase sections to more closely align with their own findings. Factors such as data is from a single center and all Beijing lineage isolates present should be noted in the limitations.

Responses:

We thank the reviewer for this thoughtful comment and agree that our original wording was broader than warranted by the scope of the present study. Our data were generated from a single-center cohort, and all sequenced discrepant isolates belonged to the East Asian/Beijing lineage. Therefore, the findings should be interpreted with caution and should not be generalized beyond the study setting without further validation.

In the revised manuscript, we have moderated the relevant statements in the Abstract, Discussion, and Conclusion. Rather than broadly advocating routine implementation, we now emphasize that WGS may be particularly useful as an adjunctive tool in cases of phenotypic discordance or when rifampicin resistance is strongly suspected despite inconclusive phenotypic results. We have also

strengthened the limitations section by explicitly noting the single-center design, the small number of sequenced isolates, and the fact that all sequenced isolates belonged to the Beijing lineage, which may limit the generalizability of our observations.

Minor comments:

Comment 5: rifampicin and rifampin are used interchangeable. Please be consistent.

Responses:

Thank you for noting this inconsistency. We have carefully revised the manuscript to ensure consistent use of rifampicin throughout the text, except where rifampin appears in the titles of cited references or other fixed source-specific terminology.

Comment 6: UI is not defined in the introduction.

Responses:

Thank you for pointing this out. We have now defined UI as uncertainty interval at its first appearance in the Introduction.

Comment 7: Reference 12 and 21 appear to be duplicate references.

Responses:

We thank the reviewer for identifying this duplication. We have carefully checked the reference list, removed the duplicate entry, and updated the reference numbering accordingly.

Comment 8: line 68 "pDST, which based on growth ..." should be "which is based on the growth ..."

Responses:

Thank you for catching this error. We have corrected the sentence in the revised manuscript.

Comment 9: line 195 "265 MTB isolates which tested for RIF" should be "265 MTB isolates which were tested for RIF".

Responses:

Thank you. We have corrected this grammatical error in the revised manuscript.

Comment 10: There are no definitions for the outcomes achieved.

Responses:

We thank the reviewer for this helpful comment. In the revised manuscript, we have clarified the relevant outcome definitions and descriptions to improve readability and to make the interpretation of phenotypic discordance, repeat testing results, and sequencing findings more explicit.

Re: Spectrum03261-25R1 (**Phenotypic discordance in rifampicin resistance detection among *Mycobacterium tuberculosis* isolates from China: insights from whole genome sequencing and a structured literature review**)

Dear Dr. Mao-Shui Wang:

Your manuscript has been accepted, and I am forwarding it to the ASM production staff for publication. Your paper will first be checked to make sure all elements meet the technical requirements. ASM staff will contact you if anything needs to be revised before copyediting and production can begin. Otherwise, you will be notified when your proofs are ready to be viewed.

Data Availability: ASM policy requires that data be available to the public upon online posting of the article, so please verify all links to sequence records, if present, and make sure that each number retrieves the full record of the data. If a new accession number is not linked or a link is broken, provide production staff with the correct URL for the record. If the accession numbers for new data are not publicly accessible before the expected online posting of the article, publication may be delayed; please contact ASM production staff immediately with the expected release date.

Publication Fees: For information on publication fees and which article types have charges, please visit our website. We have partnered with Copyright Clearance Center (CCC) to collect author charges. If fees apply to your paper, you will receive a message from no-reply@copyright.com with further instructions. For questions related to paying charges through RightsLink, please contact CCC at ASM_Support@copyright.com or toll free at +1-877-622-5543. CCC makes every attempt to respond to all emails within 24 hours.

ASM Membership: Corresponding authors may join or renew ASM membership to obtain discounts on publication fees. Need to upgrade your membership level? Please contact Customer Service at Service@asmusa.org.

Embargo Policy: A press release may be issued as soon as the manuscript is posted on the Spectrum Latest Articles webpage. The corresponding author will receive an email with the subject line "ASM Journals Author Services Notification" when the article is available online.

The ASM Journals program strives for constant improvement in our submission and publication process. Please tell us how we can improve your experience by taking this quick Author Survey.

Thank you for submitting your paper to Spectrum.

Sincerely,
Michael Whitfield
Editor
Microbiology Spectrum
