# Supplementary material for: Phenotypic discordance in rifampicin resistance detection among Mycobacterium tuberculosis isolates from China: insights from whole-genome sequencing and a structured literature review
Source: Microbiol Spectr. 2026 Jul 6;14(8):e03261-25. doi: 10.1128/spectrum.03261-25 (PMC13435698; doi:10.1128/spectrum.03261-25)
Supplement: Supplementary file 2 [file spectrum.03261-25-s0004.docx]

**Search strategy and screening workflow for the literature review**

1. Searching date:

July 1^th^, 2025.

2. PubMed (n=1058)

Searching syntax:

(rifampicin OR rifampin OR RIF OR RMP) AND (resistance OR resistant OR susceptibility OR susceptible OR sensitive OR sensitivity) AND rpoB AND (mutation* OR wild OR sequence OR sequencing) AND (("drug susceptibility" OR "drug resistance" OR "drug sensitivity") AND (test* OR assay*))

3. Embase (Elsevier; n=1097)

Searching syntax:

(rifampicin OR rifampin OR RIF OR RMP) AND (resistance OR resistant OR susceptibility OR susceptible OR sensitive OR sensitivity) AND rpoB AND (mutation* OR wild OR sequence OR sequencing) AND (('drug susceptibility' OR 'drug resistance' OR 'drug sensitivity') AND (test* OR assay*))

4. Web of Science (n=1584)

Searching syntax:

(rifampicin OR rifampin OR RIF OR RMP) AND (resistance OR resistant OR susceptibility OR susceptible OR sensitive OR sensitivity) AND rpoB AND (mutation* OR wild OR sequence OR sequencing) AND (("drug susceptibility" OR "drug resistance" OR "drug sensitivity") AND (test* OR assay*))

5. Scopus (basic search; n=1287)

Searching syntax:

(rifampicin OR rifampin OR RIF OR RMP) AND (resistance OR resistant OR susceptibility OR susceptible OR sensitive OR sensitivity) AND rpoB AND (mutation* OR wild OR sequence OR sequencing) AND (("drug susceptibility" OR "drug resistance" OR "drug sensitivity") AND (test* OR assay*))

6. Supplementary Figure. Flow diagram of included studies
